# China public emotion analysis under normalization of COVID-19 epidemic: Using Sina Weibo

**DOI:** 10.3389/fpsyg.2022.1066628

**Published:** 2023-01-09

**Authors:** Fa Zhang, Qian Tang, Jian Chen, Na Han

**Affiliations:** ^1^Department of Management Science and Engineering, Business School, Beijing Institute of Technology, Zhuhai, China; ^2^Research Base of Cross-Border Flow Risk and Governance, Beijing Institute of Technology, Zhuhai, China

**Keywords:** public, sentiment, emotion, Sina Weibo, COVID-19, China

## Abstract

The prevention and control of the coronavirus disease 2019 (COVID-19) epidemic in China has entered a phase of normalization. The basis for evaluating and improving public health strategies is understanding the emotions and concerns of the public. This study establishes a fine-grained emotion-classification model to annotate the emotions of 32,698 Sina Weibo posts related to COVID-19 prevention and control from July 2022 to August 2022. The Dalian University of Technology (DLUT) emotion-classification system was adjusted to form four pairs (eight categories) of bidirectional emotions: good-disgust, joy-sadness, anger-fear, and surprise-anticipation. A lexicon-based method was proposed to classify the emotions of Weibo posts. Based on the selected Weibo posts, the present study analyzed the Chinese public's sentiments and emotions. The results showed that positive sentiment accounted for 51%, negative sentiment accounted for 24%, and neutral sentiment accounted for 25%. Positive sentiments were dominated by good and joy emotions, and negative sentiments were dominated by fear and disgust emotions. The proportion of positive sentiments on official Weibo (accounts belonging to government departments and official media) is significantly higher than that on personal Weibo. Official Weibo users displayed a weak guiding effect on personal users in terms of positive sentiment and the two groups of users were almost completely synchronized in terms of negative sentiment. The linear discriminant analysis (LDA) was performed on the two negative emotions of fear and disgust in the personal posts. The present study found that the emotion of fear was mainly related to COVID-19 infection and death, control of people with positive nucleic acid tests, and the outbreak of local epidemic, while the emotion of disgust was mainly related to the long-term existence of the epidemic, the cost of nucleic acid tests, non-implementation of prevention and control measures, and the occurrence of foreign epidemics. These findings suggest that Chinese attitudes toward epidemic prevention and control are positive and optimistic; however, there is also a notable proportion of fear and disgust. It is expected that this study will help public health administrators to evaluate the effectiveness of possible countermeasures and work toward precise prevention and control of the COVID-19 epidemic.

## 1. Introduction

The coronavirus disease 2019 (COVID-19) pandemic poses a serious threat to human life and health (WHO, 2020; Mallah et al., 2021). Globally, COVID-19 has impacted not only people's physical health (Sohail et al., 2022; Yu et al., 2022a) but also their mental health (Holmes et al., 2020). The pandemic has been going on for more than two years, and localized outbreaks take place from time to time, significantly affecting people's mental health (Ren and Guo, 2020; Cui et al., 2022). Epidemic prevention and control measures, such as wearing masks, nucleic acid testing, vaccination, quarantine, and lockdowns, also have a negative impact on people's mental health (Budimir et al., 2021; Jin et al., 2021; Lin and Fu, 2022).

In China, the COVID-19 epidemic has entered a normalization phase, with a variety of prevention and control measures. In this normalization phase, how widespread are negative emotions among the Chinese public? What are the primary negative emotions? What are the causes of these negative emotions? Answering these questions is important for evaluating and improving prevention and control strategies. Sina Weibo, a microblog, is the most influential social media platform in China, and it is an important venue for the public to express their opinions and emotions. Using the emotion analysis and topic mining on Weibo, we can understand the emotions and concerns of the public and may therefore be able to help improve policies for the prevention and control of the COVID-19 epidemic.

Since the outbreak of the COVID-19 epidemic, researchers have analyzed social media and found that the epidemic has had a negative psychological impact on the public (Tan et al., 2021; Wang J. et al., 2022). Studies found that the factors that affect public sentiment include the number of COVID-19 cases (Da and Yang, 2020), gender (Naseem et al., 2021), and high-impact users (Xie et al., 2021). Prevention and control measures also have an impact on people's psychology. The subtypes of prevention and control strategies have different effects on sentiments and concerns (Sukhwal and Kankanhalli, 2022). The relationship between epidemic prevention measures and people's sentiments is complex Chum et al. (2021). Ogbuokiri et al. (2022) found that, in three different South African cities, citizens had different attitudes toward vaccines.

The sentiment analysis of social media can serve as a monitoring tool (Crocamo et al., 2021) and can help us understand public sentiment. However, it is not sufficient to understand emotions and their reasons. Emotion is a fine-grained sentiment and has an important impact on human behavior and decision-making (Hockenbury et al., 2016). There are various theories of emotion in psychology (Brady, 2021). Some psychologists believe that humans experience several basic emotions (Levenson, 2011). Ekman (1992) proposed six emotions: joy, sadness, anger, fear, disgust, and surprise. Plutchik (2001) proposed the emotion wheel model, which suggests that there are eight (four pairs) two-way emotions: joy and sadness, anger and fear, trust and disgust, and surprise and anticipation. The Ortony, Clore, and Collins (OCC) model defines 22 classes of basic emotions (Ortony et al., 1990). Parrott (2000) proposed a three-layer emotion model that has six primary emotions: love, joy, surprise, anger, sadness, and fear. Other psychologists argued that emotions are not discrete and have proposed a dimensional emotion model. The most influential are Russell's two-dimensional (2D) annular model and Plutchik's parabolic cone space model. Recently, Cowen extracted 27 emotions based on self-reports and believed that emotions were not clearly separated but gradually changed (Cowen and Keltner, 2017).

Social media primarily uses text and symbols. The emotion analysis of a text adopts a category emotion model that defines several basic emotions (Acheampong et al., 2020). Several studies have conducted emotion analyses on social media using different emotion-classification methods. Oliveira et al. (2022) analyzed tweets labeled with the emotions of anger, sadness, optimism, and joy. Ye et al. (2020) adopted Ekman's six basic emotions. Shen et al. (2021) adopted four categories of emotion: anger, fear, encouragement, and hope. Shi et al. (2022) classified emotions into seven categories. Wang H. et al. (2022) used the DLUT sentiment ontology, which includes seven types of emotions.

Text emotion analysis is a multi-classification problem (Murthy and Kumar, 2021). A lexicon-based approach can achieve high accuracy if the lexicon is complete, and the rules are well designed. In recent years, machine learning and deep learning models, such as Recurrent Neural Network (RNN), Long-Short Term Memory (LSTM), and Bidirectional Encoder Representations from Transformers (BERT), have also been used for text emotion analysis (Xu et al., 2020; Yu et al., 2022b). The commonly used BERT, a pre-trained model that needs to be fine-tuned on downstream tasks, can obtain better classification results (Acheampong et al., 2021). There are some BERT models pre-trained for the Chinese corpus, but it is difficult to fine-tune them due to the lack of a fine-grained emotion-labeled Chinese Weibo corpus (Xu et al., 2020). The lexicon-based method depends mainly on the lexicon and rules. English emotion lexicons, such as WorldNet-Affect (Strapparava and Valitutti, 2004), EmoSenticNet (Poria et al., 2014), and NRC word-emotion lexicons (Mohammad and Turney, 2013), are mature. The Chinese lexicons mainly include the HowNet, NTUSD, and Boson NLP. However, these lexicons provide only the positive and negative polarities of words. The DLUT sentiment ontology library adds “good” to Ekman's six basic emotions, and the emotions are divided into seven categories (Xu et al., 2008) that also give the intensity for each word. However, the DLUT has not been updated for a long time, lacks popular words from the Internet, and has some defects in emotional classification.

In summary, a few previous studies focused on the distribution of people's emotions during the normalization of the COVID-19 epidemic. The emotion classification of Chinese Weibo was also not reasonable. The lexicon-based sentiment classification method does not consider the synthesis of multiple sentences in microblogs. A few studies have analyzed whether official microblogs play a role in guiding public emotions.

This study aimed to explore the sentiments, emotions, and concerns of the Chinese public during the normalization phase of the COVID-19 prevention and control. First, a web crawler was used to obtain Weibo posts related to the prevention and control of COVID-19, and the data were preprocessed. Then, this study proposed an emotion annotation method that considers the role of sentences in different positions and the relationship between the emotion word and its modifiers. Finally, this study presented the distribution of sentiments and emotions of the Chinese public, analyzed the relationship between official and personal users, and performed LDA topic mining on negative emotion posts to understand public concerns. It is hoped that this study will help improve the policy of COVID-19 prevention and control and alleviate the negative emotions of the public.

## 2. Materials and methods

### 2.1. Data acquisition

During the normalization phase of the COVID-19 epidemic prevention and control, China adopted a dynamic clearing strategy, with different measures implemented in different regions according to each local situation. From July 2022 to August 2022, most of China was free of the epidemic, but a few regions such as Xinjiang, Hainan, and Tibet had localized epidemic, which is a typical scenario during the normalization phase.

Weibo posts related to COVID-19 prevention and control were collected using the Sina Weibo search template in the Octopus web crawler. The keywords were set as “New Coronavirus” OR “epidemic” OR “COVID-19” OR “prevention and control.” Because the Sina Weibo search engine provides only 50 pages of results at a time, this study conducted multiple data crawls, combined the results, de-duplicated them according to the publisher and content, and finally obtained a total of 32,698 posts. The fields of the dataset included publisher, content, publisher link, posting time, source, number of comments, number of retweets, number of likes, and current time.

### 2.2. Data preprocessing

Some fields in the dataset could not be used directly, and there was an abundance of irrelevant content in the posts that needed to be cleaned. In this study, Python programs were written to preprocess the data, such as the calculation of the posting date and time, user ID extraction, content cleaning, and user type determination.

The “posting time” in the original dataset is represented by “*x seconds ago*, “*x minutes ago*,” and “*Today HH:MM*.” In this study, the date and time of posting, based on the fields “current time” and “posting time., were calculated.

Due to privacy protection measures, the publishers are anonymous. To obtain basic information about the user, their user ID was extracted from the field “publisher link” using regular expression. For example, if the publisher link was https://weibo.com/7412756093?refer_flag=1001030103, the user ID “*7412756093*” was parsed using Python package re.

A considerable amount of content was mentioned in the Weibo posts that was not related to the sentiment analysis and had to be cleaned. In this study, irrelevant content, such as Weibo topic, user name, and URL (Uniform Resource Locator) using regular expressions, was eliminated and then stop words removed using the Baidu stop words dictionary.

Weibo users were divided into two categories: official users and personal users. Posts released by official media and government departments are often announcements on epidemic and policy propaganda. Official media includes television (TV) stations, radio stations, newspapers, magazines, and portal websites. Government departments, such as the Health and Welfare Commission and CDC, often publish Weibo posts as well. To determine the type of user, basic user information is crawled according to the user ID. The user dataset contains fields such as “username,” “brief description,” “certification,” and “industry.” The user type can be inferred from the fields “username” and “brief description.” In this study, a list of keywords that could be used to describe official users was defined. A Python program uses keyword-related regular expressions to find official users and then correct them manually.

After preprocessing, the fields of the dataset include publisher, user ID, user type, content, cleaned content, posting date, posting time, posting source, number of comments, retweets, and likes.

### 2.3. Emotion classification

Weibo posts may be long and may contain several sentences. First, the post is split into sentences using separators “. °! ?”. Sentences in different positions may play different roles in expressing emotions, and the first and last sentences may be more important. If the number of sentences *n* ≤ 2, let the weight of each sentence $w_{i}=\frac{1}{n},1\leq i\leq n$; otherwise, for *n*>2, the weights of the first and last sentences are increased, and the weight of each sentence is calculated as follows:


$$\begin{array}{l} w_{i}=\{\begin{array}{c} \frac{2}{n+2}\;,\;i=1,n\;\\ \frac{1}{n+2}\;,\;i=2,\ldots ,n-1\; \end{array} \end{array}$$

An emotional score was calculated for each sentence. The sentence was matched with the emotion lexicon to extract the emotion word, and the emotion type of the sentence was determined by the emotion type of the emotion word. Dependent syntactic analysis was performed with the emotion word as the central word to obtain the emotion unit. The emotion unit is centered on the emotion word and may contain some degree of adverbs, negation words, and gerunds. Assuming that the type of emotion word is *j*, its intensity is *q*, the number of negation words is *m*, the number of degree adverbs or gerunds (which play an enhancing or weakening role) is *l*, and the adjustment factors for degree adverbs or gerunds are *c*_*v*_, *v* = 1, 2, ..., *l*, then, the score for emotion type *j* is calculated as follows:


$$\begin{array}{l} e_{j}={\left(-1\right)}^{m}\overset{l}{\underset{v=1}{\prod }}c_{v}q_{\;} \end{array}$$

The emotion unit is scored 0 for other emotion types.

One or more negative words may be present in a sentence, due to which the emotion score may be negative, and therefore needs to be converted to reverse emotion. However, seven emotions are mentioned in the DLUT ontology, among which good and disgust, happy and sadness, and anger and fear can be combined into two-way emotions, but there is no reverse emotion for the emotion of surprise. According to Plutchik's emotion wheel model, if the emotion type is surprise and the intensity score is negative, it is converted to anticipation. Therefore, emotions were divided into eight categories (four pairs): good-disgust, joy-sadness, anger-fear, and surprise-anticipation.

If there are multiple emotion units in a sentence, each with a different emotion type, the scores of all emotion units for each type of emotion are combined to obtain the emotion vector of the sentence as follows:


$$\begin{array}{l} E_{i}=\left(e_{i1},e_{i2},\ldots ,e_{i8}\right) \end{array}$$

If the Weibo post contains multiple sentences, the emotion vector of each sentence is weighted and summed up to obtain the emotion vector of the post as follows:


$$\begin{array}{l} 𝔼=\overset{\;}{\underset{i}{\sum }}w_{i}E_{i}=\left(E_{1},E_{2},\ldots ,E_{8}\right) \end{array}$$

The emotion type with the highest score in the vector 𝔼 was selected as the emotion type of the post, $k=\underset{j}{argmax}\{E_{j}\}$. If the maximum score $E_{k}<\delta$, where δ is a predefined threshold, the emotion is classified as neutral.

### 2.4. Evaluation of the model

The present study randomly selected 2,000 posts from the Sina Weibo corpus for manual annotation to determine the sentiment and emotion. The emotion-classification algorithm explained was applied to the test set, and the performance metrics of the model were obtained by comparing its predictions with the annotated labels.

For sentiment classification, the accuracy of the model was 0.80. Performance metrics such as precision, recall, and F1-score are shown in Table 1. This is a multi-classification problem, and in this study macro-averaging was used to obtain the overall performance. The macro-average is an arithmetic average of the performance of each category. The macro precision was 0.83, macro recall was 0.79, and macro F1-score was 0.79.

**Table 1:** Performance metrics of the algorithm in terms of sentiment classification.

**Sentiment**	**Precision**	**Recall**	**F1-score**
Positive	0.70	0.98	0.82
Neutral	0.86	0.66	0.74
Negative	0.93	0.73	0.82
Macro average	0.83	0.79	0.79

The accuracy of the model for emotion classification was 0.78. The performance metrics of precision, recall, and F1-score are listed in Table 2. The macro precision was 0.81, macro recall was 0.66, and macro F1-score was 0.69.

**Table 2:** Performance metrics of the algorithm in terms of emotion classification.

**Emotion**	**Precision**	**Recall**	**F1-score**
Good	0.71	0.91	0.79
Joy	0.58	0.97	0.73
Surprise	0.92	0.67	0.77
Anticipate	0.67	0.11	0.19
Neutral	0.86	0.66	0.74
Disgust	0.94	0.68	0.79
Fear	0.94	0.85	0.89
Anger	0.73	0.45	0.56
Sadness	0.92	0.67	0.78
Macro average	0.81	0.66	0.69

### 2.5. Topic mining

Several popular topic models, including Latent Semantic Analysis (LSA), Probabilistic Latent Semantic Analysis (PLSA), and Latent Dirichlet Allocation (LDA). The LSA model is simple but lacks a solid foundation in mathematical statistics and requires Singular Value Decomposition (SVD) operations, which are inefficient. PLSA introduces a probabilistic model and uses the maximum likelihood for solving. However, the parameters to be estimated increase linearly with document size. The LDA is a model proposed by Blei et al. (2003). The LDA is based on the Bayesian theory, with a clear internal structure, and a number of parameters that is independent of the corpus; it is suitable for large-scale corpora. In the present study, LDA topic mining was implemented using the gensim (Ehek and Sojka, 2010) package in Python and extracted public concerns from the Weibo posts. LDA is a widely used topic mining method; however, it is difficult to determine the optimal number of topics. Blei et al. suggested selecting the number of topics with a minimal amount of perplexity. However, the larger the number of topics, the smaller the perplexity, which often leads to a very large optimal number of topics. Some scholars have used the elbow method to select the number of topics with an obvious turn in perplexity as the optimal number. Another frequently used metric is coherence, which measures the semantic similarity of keywords within the same topic. The greater the coherence, the higher the cohesion within the topic. However, coherence does not reflect whether different topics can be separated sufficiently.

The present study used both perplexity and coherence, combined with LDA visualization, to determine the optimal number of topics. First, the maximum number of topics was determined using the perplexity elbow method. Then, the possible optimal number of topics was selected based on coherence. Finally, the optimal number of topics was determined by observing the separation of topics using pyLDAvis (Python library for interactive topic model visualization) (Sievert and Shirley, 2014).

## 3. Results

### 3.1. Public sentiments and emotions

Emotions were annotated for the 32,698 Weibo posts. The proportion of each emotion was then calculated. To perform sentiment analysis, this study categorized the four emotions, good, joy, surprise, and anticipation, as positive sentiments, and categorized the other four emotions, fear, disgust, sadness, and anger, as negative sentiments. The sentiment and emotion distribution of Weibo posts during the epidemic normalization phase is shown in Table 3.

**Table 3:** Sentiment and emotion distribution of China public.

**Sentiment/emotion**	**Number of posts**	**Percentage (%)**
**Positive**		
Good	12,515	38.27
Joy	3,965	12.13
Surprise	109	0.33
Anticipate	30	0.09
**Neutral**	8,296	25.37
**Negative**		
Fear	3,633	11.11
Disgust	2,635	8.06
Sadness	889	2.72
Anger	626	1.91

Positive sentiments accounted for 50.83% of the posts, neutral for 25.37%, and negative for 23.80%. It not only showed that more than 50% of the Weibo posts express positive sentiments, but also showed that close to one-fourth express negative sentiments, which is worth noting. The main emotions in positive sentiments were good (38.27%) and joy (12.13%), indicating that many Internet users were satisfied and optimistic about epidemic prevention and control. The main emotions in negative sentiments were fear (11.11%) and disgust (8.06%), indicating that some Internet users express fear and disgust, while a small number of them feel sadness (2.72%) and anger (1.91%).

### 3.2. Sentiment difference between official Weibo and personal Weibo

During the normalization phase of the COVID-19 prevention and control, the government takes responsibility for epidemic prevention and control, and the official media complies with reporting norms, becoming more cautious when publishing on Weibo. Personal users are free to publish and express their emotions and opinions directly. In this study, all posts were divided into two groups based on the type of publisher: official Weibo posts and personal Weibo posts. There were 6,411 official posts and 26,155 personal posts (a small number of posts were excluded due to difficulty in determining the type of publisher). A sentiment analysis was performed on the two groups of posts, and the sentiment and emotion distribution of the two groups is shown in Table 4.

**Table 4:** Sentiment and emotion distribution of official Weibo and personal Weibo.

**Sentiment/emotion**	**Official posts**		**Personal posts**	
	**Number of blogs**	**Percentage (%)**	**Number of blogs**	**Percentage (%)**
**Positive**				
Good	3,149	49.12	9,292	35.59
Joy	736	11.48	3,209	12.29
Surprise	3	0.05	106	0.41
Anticipate	1	0.02	29	0.11
**Neutral**	900	14.04	7,344	28.13
**Negative**				
Fear	1,176	18.34	2,448	9.38
Disgust	249	3.88	2,368	9.07
Sadness	74	1.15	809	3.10
Anger	123	1.92	500	1.92

**Figure 1:**
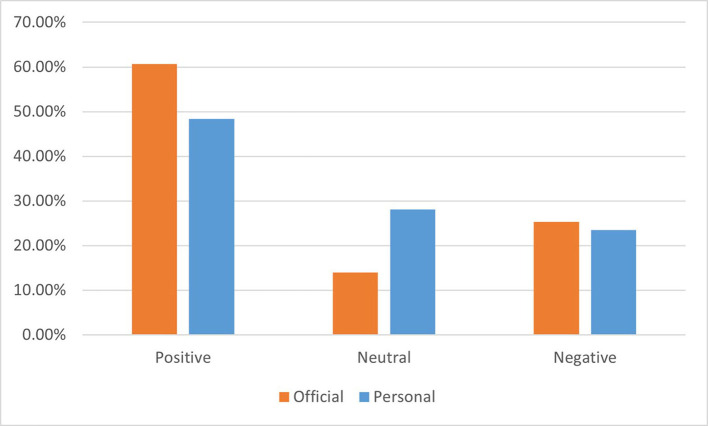
Comparison of sentiment distribution between official Weibo and personal Weibo.

On official Weibo, positive sentiment accounted for 60.7% of the posts, neutral sentiment accounted for 14% of the posts, and negative sentiment accounted for 25.3% of the posts. For personal Weibo, positive sentiment accounted for 48.4% of the posts, neutral sentiment accounted for 28.1% of the posts, and negative sentiment accounted for 23.5% of the posts. A comparison of sentiments between the two groups is shown in Figure 1. A chi-square test was conducted on the distribution of sentiments, with *P* < 0.05, indicating a significant difference between the two groups. The percentage of positive sentiments in the official group was significantly higher than that in the personal group, and the percentages of negative sentiments were similar in both groups, indicating that official users were more positive and optimistic toward the COVID-19 epidemic.

### 3.3. Guiding effect of official users

In the normalized phase of epidemic prevention and control, do official users have a guiding effect on personal users? If so, is the guiding effect strong or weak? To explore the relationship between official and personal users, two main positive emotions, good and joy, and two main negative emotions, fear and disgust, were selected in this study.

**Figure 2:**
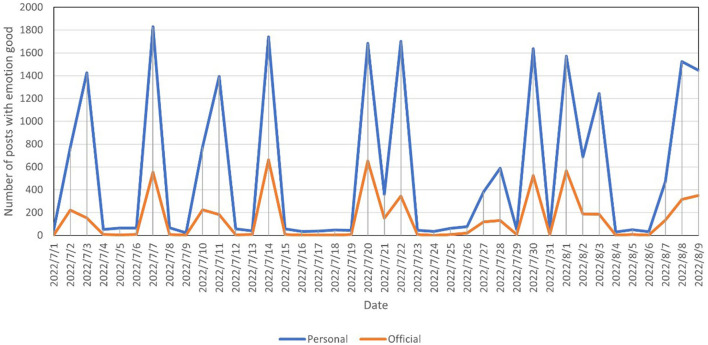
Number of blogs with the emotion of good posted by official and personal users.

**Figure 3:**
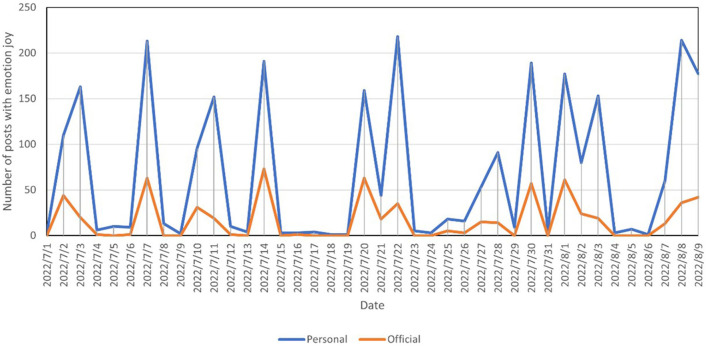
Number of blogs with the emotion of joy posted by official and personal users.

The present study counted the number of daily posts with the emotion of good on official and personal Weibo and plotted the time series, as shown in Figure 2. Similarly, the time series of daily posts with the emotion of joy is shown in Figure 3. To facilitate the comparison of trends, the number of official posts was scaled up.

Figure 2 reveals that most of the time, the trends for official and personal posts were the same, but in a few periods, official posts reached a peak before personal posts. For example, official posts reached a peak on July 2, while personal posts reached a peak on July 3. This phenomenon exists for both good and joy emotions. It is suggested that, in terms of positive sentiment, official users have a guiding effect on personal users, but the effect is weak.

**Figure 4:**
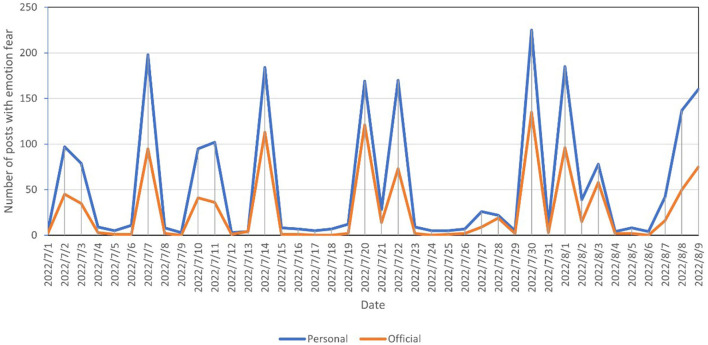
Number of blogs with the emotion of fear posted by official and personal users.

**Figure 5:**
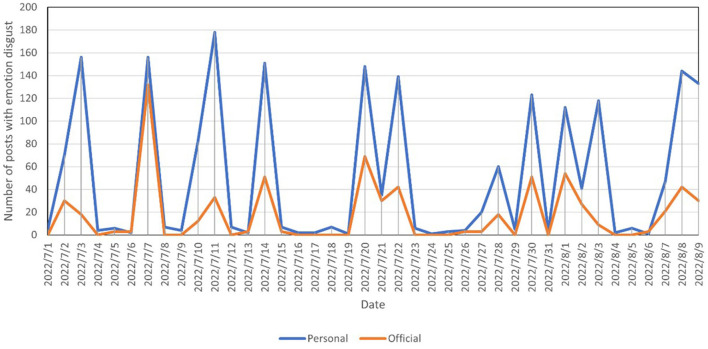
Number of blogs with the emotion of disgust posted by official and personal users.

Is there a similar phenomenon for negative emotions? In this study, the time series of official and personal posts with the emotions of fear and disgust, respectively, were plotted, as shown in Figures 4, 5, and found that trends for official and personal posts were almost identical. This is clearly different from the time series of good and joy emotions. It is presumed that when it comes to negative emotions, official users have no guiding effect on personal users.

### 3.4. Topics related to negative emotions

The percentage of negative sentiments in all of the Weibo posts was 23.80%, which is noteworthy. Negative sentiments may trigger risk events that require further analysis. Topics associated with negative sentiments are helpful for understanding public concerns. This study performed LDA topic mining on the personal posts with emotions of fear (9.38%) and disgust (9.07%), which accounted for a relatively large proportion of negative posts.

#### 3.4.1. Topics related to emotion of fear

**Figure 6:**
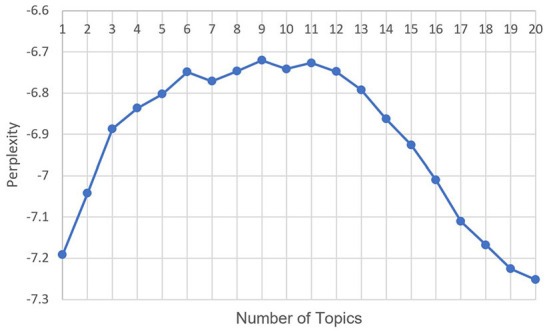
Relationship between the number of topics and the perplexity in fear posts.

**Figure 7:**
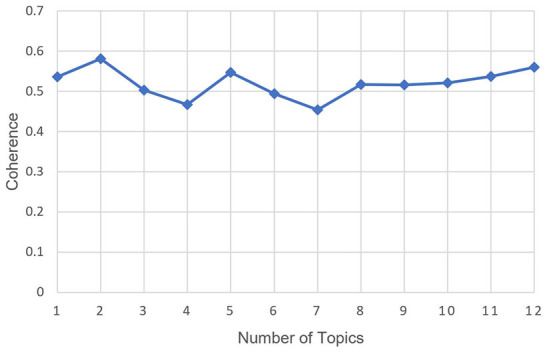
Relationship between the number of topics and the coherence in fear posts.

The number of topics in the LDA model was a hyperparameter that needed to be selected in advance. First, the topic number was set to range from 1 to 20 to obtain the relationship between the number of topics and perplexity, as shown in Figure 6. The elbow method was used to determine the upper limit of the number of topics, at 12. The coherence is then calculated for each topic number from 1 to 12. The relationship between the number of topics and the coherence is shown in Figure 7. Topic number 5, with the highest coherence, was selected for the LDA.

**Figure 8:**
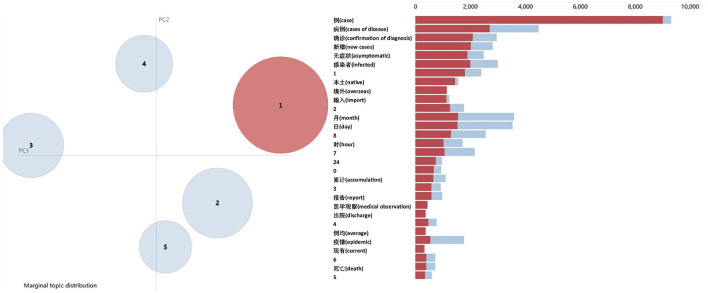
The pyLDAvis visualization results for fear posts (topic number 5).

The present study visualized the LDA results using pyLDAvis, which is a dynamic interaction graph. The principal component analysis (PCA) of the two-dimensional (2D) projections of the five topics are shown in Figure 8. These five topics are clearly separated, and their keywords are significantly different. Then, the optimal number of topics is determined as 5.

The number of topics was set to five and the LDA performed on the personal posts with the emotion of fear. The keywords for the five topics are listed in Table 5, in descending order of weight.

**Table 5:** Keywords of the five topics with emotion of fear.

**Topic**	**Keywords**
1	Virus, COVID-19, outbreak, infection, mutation, transmission, omicron, human, vaccine
2	Case, confirmed, new, infected, asymptomatic, month, day, native, foreign, imported
3	Cases, confirmed, new, COVID-19, vaccination, million, vaccine, cumulative, death, Korea, infection
4	Positive, infected, test, nucleic acid, found, personnel, control, virus, COVID-19, case, isolation
5	Case, outbreak, personnel, prevention and control, Tibet, age, asymptomatic, Yazhou District, nucleic acid

Based on the keyword list for each topic, combined with qualitative analysis of typical Weibo posts, the topics associated with the emotion of fear were refined into this list:

- COVID-19 spread and mutation.
- The increase in confirmed cases within and outside the country.
- Infection and death caused by COVID-19.
- Control of personnel with positive nucleic acid tests.
- Local outbreak prevention and control.

These five topics have a semantic overlap and therefore can be grouped further into three topics:

- Infection and death due to the COVID-19 epidemic.
- Control of personnel with positive nucleic acid tests.
- Epidemics within and outside the country, especially locally.

#### 3.4.2. Topics related to emotion of disgust

**Figure 9:**
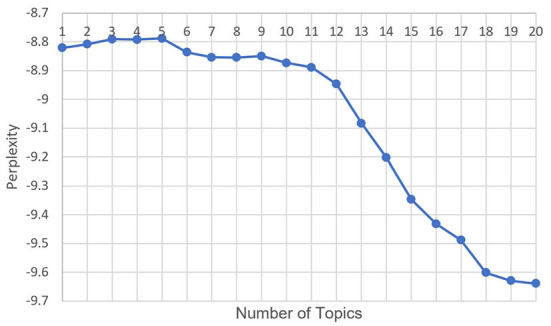
Relationship between the number of topics and the perplexity in disgust posts.

**Figure 10:**
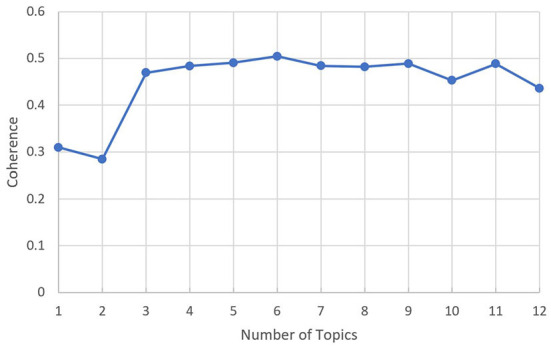
Relationship between the number of topics and the Coherence in disgust posts.

The LDA was also performed on personal posts expressing the emotion of disgust. First, the upper limit of the number of topics was set to 12 (see Figure 9). Then, the number six was selected as the optimal number of topics based on the coherence metric (see Figure 10).

**Figure 11:**
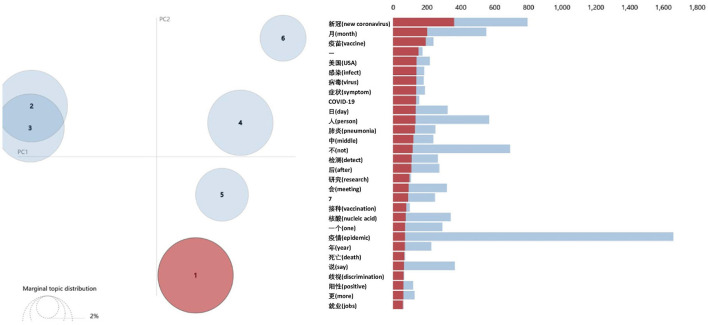
The pyLDAvis visualization results for disgust microblogs (topic number 6).

The LDA was performed with topic number six and visualized using pyLDAvis. It was found that topics 2 and 3 overlapped on the PCA projection (see Figure 11), hence they were combined into one topic, and the topic number was adjusted to 5.

The the number of topics was set to five, the LDA performed, and a list of keywords obtained for each topic, as shown in Table 6.

**Table 6:** Keywords of the five topics with emotion of disgust.

**Topic**	**Keywords**
1	Epidemic, COVID-19, bad, really, people, can't, want to say, prevention and control, nasty
2	Outbreak, prevention and control, nucleic acid, personnel, testing, work, charges, isolation, COVID-19
3	COVID-19, infectious disease, someone, crime, control, epidemic, obstruction, suspected, fever
4	COVID-19, vaccine, detection, U.S., symptoms, infection, virus, human, pneumonia
5	Prevention and control, outbreak, charges, nucleic acid, remediation, personnel, found, several, not, implemented, people

According to the keywords of each topic, combined with typical Weibo posts, for qualitative analysis, the five topics associated with disgust were extracted as follows:

- Disgust with the outbreak, and the prevention and control of the epidemic.
- Nucleic acid testing charges.
- Someone is suspected of a crime that hinders the prevention and control of the epidemic.
- Infections outside the country, especially in the United States of America.
- Failure to implement epidemic prevention and control measures.

Summarizing these five topics further, the topics related to disgust are:

- Persistence of the COVID-19 epidemic.
- Non-compliance and non-implementation of prevention and control measures.
- Costs for nucleic acid testing.
- Outbreak of COVID-19 outside the country.

## 4. Discussions

The present study collected Sina Weibo posts during the normalization phase of the COVID-19 epidemic prevention and control. A fine-grained emotion-classification algorithm was designed with consideration given to the positions of sentences and syntactic structure of the emotion unit. The algorithm was used to label the emotions of the Weibo posts. Based on the labeled data, the present study analyzed the sentiments of the Chinese public. The outcome showed that most of the population was positively optimistic about the epidemic, with good and joy being the main emotions. However, there is also a certain proportion of negative sentiment, with the main negative emotions being fear and disgust. The LDA topic mining was conducted on personal Weibo posts containing negative emotions. The results showed that the negative emotions of the public were mainly related to the long-term existence of the epidemic, control of personnel with positive nucleic acid test results, non-implementation of prevention and control measures, and local outbreaks.

In this study, a lexicon-based emotion-classification method was developed. The emotion ontology of DLUT was expanded to form four pairs of bidirectional emotions. It solved the problem that the DLUT emotion system cannot handle when the emotion is surprise, and when the emotion's score is negative. This emotion-classification system may be useful for emotion analyses.

The present study used the LDA for topic analysis of negative sentiment posts. The LDA is a popular topic-mining method (Xie et al., 2021; Ogbuokiri et al., 2022; Oliveira et al., 2022; Wang H. et al., 2022). However, it was difficult to determine the optimal number of topics. Ogbuokiri et al. (2022) used Jaccard similarity to compare the similarity between two topics and then filtered the topics based on the metric of coherence. Due to the high dimensionality of the text data, results may differ when different thresholds are used. The present study used perplexity and coherence to initially select the number of topics, and then determined the optimal number of topics using pyLDAvis. This approach ensures a high degree of semantic consistency within topics as well as a good degree of topic separation.

The present study showed that positive sentiment accounted for more than 50% of the total sentiment between July 2022 and August 2022. Pan et al. (2021) studied the comment data of the People's Daily account on Sina Weibo, where positive sentiments accounted for more than 50%, which is close to our result. Wang J. et al. (2022) showed that when the World Health Organization (WHO) declared a global pandemic on 11 March 2020, the sentiment score of the global population dropped significantly, dominated by negative sentiments, but largely recovered to pre-pandemic levels in May 2020. Tan et al. (2021) reported similar findings, with a significant drop in public sentiment during the Wuhan 2020 epidemic. However, after the Wuhan 2020 epidemic ended, the overall sentiment of Chinese Weibo users recovered (although not to pre-epidemic 2020 levels), with sentiment scores fluctuating around 0.5, and with no correlation to new cases. These studies imply that despite the persistence of the epidemic, public sentiment gradually recovered after the end of the outbreak. The COVID-19 epidemic has now existed for more than 2 years, and with no serious outbreaks from July 2022 to August 2022, people's psychological resilience has largely recovered.

The present study showed that official users had a significantly higher positive sentiment than personal users and that official posts had a weak guiding effect on personal users in terms of positive sentiment. Some studies have focused on the roles of different users in social media. Pan et al. (2021) revealed that big V users were more important. However, there are not many studies on the interaction between official users and personal users. The government and official media play a very important role in epidemic prevention and control. Our study could help the government evaluate and improve the role and value of official media.

The present study showed that nearly one-fourth of the Weibo posts contained negative emotions, mainly fear and disgust. Fear was mainly related to infections and deaths due to the epidemic, the presence of the epidemic, and the control of people who tested positive for nucleic acids. Some other studies also noted that social media users expressed fear during the epidemic, but did not analyze the reasons behind the emotion. Oliveira et al. (2022) analyzed English Twitter feeds from 12 countries from January 2020 to April 2021, and found that the main negative emotions were fear and sadness. Wang H. et al. (2022) studied public sentiment related to the Omicron variant on Sina Weibo and found that fear accounted for the largest proportion of negative emotions. These studies suggest that fear may be one of the more important negative emotions during an epidemic. A few studies have focused on the emotion of disgust during epidemics. The present study revealed the percentage of disgust emotions and the topics related to disgust. Due to the prolonged existence of the COVID-19 epidemic, public disgust with epidemics is an important and noteworthy phenomenon. Failure of public health authorities to alleviate the disgust of the population may lead to the failure of prevention and control measures.

## 5. Conclusions

In this study, Sina Weibo posts were collected during the normalization phase of the COVID-19 epidemic prevention and control. The present study conducted an emotion analysis of the Weibo posts, which can reveal people's psychological feelings in more detail than a sentiment analysis. The present study improved the emotion-classification system of the DLUT and developed a lexicon-based emotion-classification method. During the normalization of the COVID-19 epidemic, more than 50% of the Weibo posts had positive sentiments, but more than 20% of the Weibo posts had negative sentiments. In terms of positive emotions, government departments and official media played a role in guiding personal users, but this effect was weak. The negative emotions were mainly fear and disgust, which were related to topics such as the persistence of the epidemic, control of nucleic acid-positive persons, non-implementation of prevention and control measures, and domestic and international outbreaks. This study can help public administrators understand public emotions and evaluate policy in a timely manner. The results of the study, such as the main negative emotions and related topics, provide clues to understand public concerns, which can help to adjust policies and achieve more effective prevention and control measures.

The present study has some limitations. First, the sentiment lexicon has not been updated in recent years because the online language has been changing rapidly. It is planned to collect buzz words from social media and update the lexicon. Second, there are texts, emojis, and even pictures and videos in Weibo posts, and integrating multiple types of media can be considered to determine emotions in the future. Finally, the topics obtained through LDA can only provide clues for understanding sentiments. To discover the mechanisms behind these sentiments, text mining must be combined with statistical analysis, interviews, experimental studies, etc.

## Data availability statement

The original contributions presented in the study are included in the article/supplementary material, further inquiries can be directed to the corresponding author.

## Author contributions

FZ contributed to the conception, design, analysis, and revision of the manuscript. QT contributed to data collection and analysis. JC contributed to the results and discussions. NH contributed to sentiment analysis programming. All authors contributed to the article and approved the submitted version.
